# The Creation of the Cardiac Society of Timor-Leste: A Major Step Forward in Timorese Cardiac Health

**DOI:** 10.5334/gh.1560

**Published:** 2026-06-04

**Authors:** Elizabeth D. Paratz, Rachel Nunn, Jane Williams, Zoe Fitzgerald, Diana Marques, Sidonia Ximenes, Ricardo Flavio, Claudia Natalicia Magno, Jacinto da Costa Vinhas, Sidonio Viana, Cesaltinho Leão, Waldo Milian, Alan Appelbe, Virag Kushwaha, Simon Eggleton, Louise Creati, Will Wilson, Noel Bayley, David Lloyd, Herculano Seixas dos Santos

**Affiliations:** 1 St Vincent’s Hospital Melbourne, 41 Victoria Parade, Fitzroy VIC 3065, Australia; 2 St Vincent’s Institute of Medical Research, 9 Princes St, Fitzroy VIC 3065, Australia; 3 Timor-Leste Hearts Foundation, Docklands, Melbourne VIC 3005, Australia; 4 Department of Cardiology, Hospital Nacional Guido Valadares, Estrada de Bidau, Dili, Timor-Leste; 5 Department of Cardiology, Barwon Health, 272–322 Ryrie St, Geelong VIC 3220, Australia; 6 Department of Cardiology, Eastern Heart Clinic, Level 3, Prince of Wales Hospital, Barker St, Randwick, Sydney NSW 2031, Australia; 7 Department of Cardiology, Royal Melbourne Hospital, 300 Grattan St, Melbourne VIC 3000, Australia; 8 Department of Cardiology, Warrnambool Hospital, 25 Ryot St, Warrnambool VIC 3280, Australia

**Keywords:** global health, rheumatic heart disease, Timor-Leste, non-communicable diseases, Asia-Pacific

## Introduction

In 2002, Timor-Leste became the first new sovereign state of the 21st century, and in 2025 its national cardiac society became the newest member of the World Heart Federation. Timor-Leste is a small tropical island one hour north of Australia by air. Despite its location proximate to highly developed countries, it remains a low- and middle-income country (LMIC), with approximately 44% of the population living below the poverty line, as well as endemic rheumatic heart disease (RHD) and high rates of other cardiovascular diseases (CVDs) (1). In hand with the newly developed National Heart Health Strategy for 2025–2035 (2), the Cardiac Society of Timor-Leste will enable Timorese cardiac capacity building at individual, organisational and system levels.

## Cardiac Care in Timor-Leste

### Personnel and facilities

At the time of Timorese independence in 2002, there were only around 20 primary-care doctors in the country (3). The Cuban government provided pre-clinical training to around 700 doctors over the next decade, and bilateral Timor–China agreements resulted in Timorese doctors receiving training in China and more than 100 Chinese doctors providing care in Timor-Leste. In 2020, the World Bank reported that there were eight doctors per 10,000 people in Timor-Leste, the median value in LMICs being 13 doctors and in neighbouring Australia 40 doctors (4). There are currently no cardiac-trained nurses, sonographers or cardiac technicians in Timor-Leste (2).

Primary care is provided by local healthcare workers, with 76 community health centres and 333 health posts across Timor-Leste (2). Cardiac assessments may be undertaken at one of five hospitals in Baucau, Maubisse, Maliana, Suai and the enclave of Oecusse, or the tertiary Hospital Nacional Guido Valadares (HNGV) in the capital city of Dili. There is no in-country cardiac catheterisation laboratory or cardiac surgical capacity yet: development of a cardiac catheterisation laboratory within Timor-Leste (2) is planned for 2030. The Timor-Leste Hearts Fund (TLHF, previously the East Timor Hearts Fund) has been active as an official organisation since 2010 (1), and has provided and funded cardiac interventions for over 100 young Timorese, overwhelmingly in Australia (5).

## Cardiovascular Health in Timor-Leste

Major challenges in modern Timor-Leste include RHD, the impact of unrepaired congenital cardiac disease, CVD risk factor burden and environmental pollution, all underpinned by limited CVD data collection.

RHD screening studies within the school-age population have demonstrated a prevalence of approximately 3.5% (6). Amongst patients presenting to cardiology clinics, one-fifth arrive with advanced disease requiring either immediate surgery or palliation (1). In a country with a median age of 21, endemic RHD imposes a substantial burden of premature cardiovascular morbidity and mortality.

Congenital cardiac lesions typically go unrepaired, due to both a lack of diagnosis and an absence of in-country interventional capacity. Consequently, congenital cardiac disease is a major source of premature mortality and heart failure, accounting for 6% of adult cardiac presentations and 40% of paediatric presentations (3).

Timor-Leste has an elevated age-standardised CVD mortality rate that is both 1.6-fold the global average and likely under-reported. Adverse CVD risk factors include very high rates of smoking, with 45% of males aged 13–15 smoking regularly (7), and more than 25% of the adult population exhibiting hypertension (8). Protective factors are the young median age of Timorese citizens, below-average age-standardised rates of hyperlipidaemia, diabetes and obesity, and above-average rates of physical activity (8). As the Timorese population starts to age and becomes more affluent, it is likely these protective elements will be lost.

Environmental pollution is an increasingly recognised contributor to cardiovascular mortality. Household solid fuel use and second-hand smoke exposure result in Timor-Leste ranking in the top three countries globally in the rising trajectory of CVD burden attributed to particulate matter pollution (9).

Underpinning these challenges is the issue of accurate data collection. For example, death certificates are not compulsory, and they require a lodgement fee and do not need to be filled in by medical personnel if death occurs in the community (10). Efforts to improve data collection are underway, with data collection an independent pillar of the National Heart Health Strategy. It is important to note that CVD morbidity and mortality are likely to experience a pseudo-rise as more accurate data is collected; it has already been observed that CVD mortality has risen substantially since 2010, but the reality underlying this finding is uncertain (2).

## The Cardiac Society of Timor-Leste

A 10-year National Heart Health Strategy (2025–2035) has been developed by the Ministry of Health (Table 1) (2). Its core vision is to achieve a 20% reduction in premature CVD mortality by 2035, focusing on six key pillars: workforce development, national policy, development of infrastructure, health promotion activities, health service delivery and data collection improvements. The creation of the Cardiac Society of Timor-Leste is an important step in the implementation of all pillars of the National Strategy.

**Table 1:** The 2025–2035 National Heart Health Strategy of Timor-Leste.


PILLAR 1: HEALTH WORKFORCE	PILLAR 2: NATIONAL POLICY AND COORDINATION	PILLAR 3: INFRASTRUCTURE AND MEDICAL PRODUCTS	PILLAR 4: HEALTH PROMOTION	PILLAR 5: HEALTH SERVICE DELIVERY	PILLAR 6: INFORMATION AND DATA

Equip primary care with increased cardiac skills: cardiology to be a compulsory rotation. Develop a cardiac nursing programme. Scale up local cardiologist workforce. Build surgical and paediatric cardiology skills. Use of national RHD guidelines as standard.	Establish a dedicated operational CVD unit within the Ministry of Health. Implement legislative measures to reduce CVD risk. Collaborate with regional and international partners. Develop localised plans to strengthen CVD care. Establish a CVD monitoring and evaluation framework.	Ensure reliable supply chains for medicines and consumables. Create an engineering unit at HNGV. Plan for a cardiac catheterisation laboratory by 2030. Utilise technological support and innovations.	Promote smoking cessation. Improve access to nutritious foods. Reduce alcohol use. Increase heart health awareness.	Strengthen community-level screening, treatment and referral. Develop capacity in regional hospitals with cardiologist and cardiac nurse staffing. Expand advanced cardiac skillset at HNGV with paediatric cardiology division and interventional cardiology by 2030. Integrate multidisciplinary care.	Improve data collection, tracking and analysis. Develop digital health records. Promote a culture of local research and develop a CVD research framework. Deliver CVD care in all languages.


Overall mission: A 20% reduction in premature CVD mortality by 2035.

**Figure 1:**
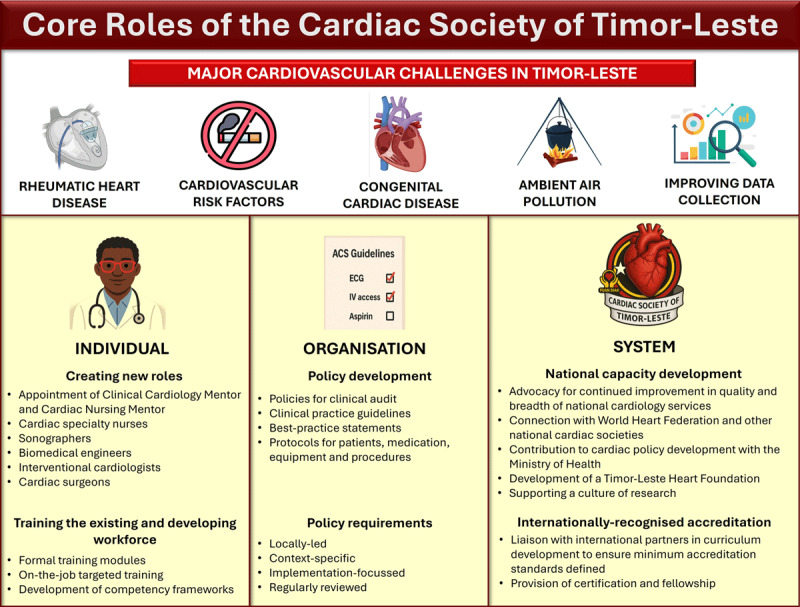
The core roles of the Cardiac Society of Timor-Leste. Capacity will be built on individual, organisation and system levels. Icons for rheumatic and congenital heart disease from BioRender.

The Cardiac Society’s role centres around strengthening individual clinician capabilities, creating organisational capacity and working at a system level to advocate for CVD-focussed policies (Figure 1).

For individuals, the Society will support in-country training pathways and a curriculum across key cardiac workforce roles, complemented by international mentoring to grow Timorese clinicians’ skillsets. All existing cardiologists will be inducted into the Society, and around 20 nurses are anticipated to undergo specialty cardiac nursing training. Additional key personnel such as sonographers, biomedical engineers, cardiac surgeons and allied health professionals will be supported over the coming years.

At an organisational level, context-specific standardised protocols will be developed for Timorese hospitals. Their development will be locally led but will benefit from international advice and mentoring.

At a system level, the Cardiac Society will advocate for Timorese cardiac health internationally, establishing clinical mentoring collaborations and benchmarking cardiac curricula to ensure a minimum standard of practice. The Cardiac Society will work closely with the Ministry of Health and partner with a future Timor-Leste heart foundation to create public health campaigns to improve cardiac care in Timor-Leste.

## Conclusion

The newly formed Cardiac Society of Timor-Leste arrives at a pivotal time in the growth of the Timorese health system. Fellows of the Society will develop a culture of continuous improvement and education in cardiac care. As the newest national society in the World Heart Federation, the Cardiac Society of Timor-Leste will gain from collaborations and mentorship from other cardiac societies that have navigated similar challenges.
